# Supplementary material for: Genomic and metabolic adaptations of biofilms to ecological windows of opportunity in glacier-fed streams
Source: Nat Commun. 2022 Apr 20;13:2168. doi: 10.1038/s41467-022-29914-0 (PMC9021309; doi:10.1038/s41467-022-29914-0)

<u>**Supplementary material**</u>

Genomic and metabolic adaptations of biofilms to ecological windows of opportunity in glacier-fed streams

Susheel Bhanu Busi[1,#], Massimo Bourquin[2,#], Stilianos Fodelianakis[2,#], Grégoire Michoud[2], Tyler J. Kohler[2], Hannes Peter[2], Paraskevi Pramateftaki[2], Michail Styllas[2], Matteo Tolosano[2], Vincent De Staercke[2], Martina Schön[2], Laura de Nies[1], Ramona Marasco[3], Daniele Daffonchio[3], Leïla Ezzat[2], Paul Wilmes[1,4,*], & Tom J. Battin[2,*]

[1]Systems Ecology Group, Luxembourg Centre for Systems Biomedicine, University of Luxembourg, Esch-sur-Alzette, Luxembourg
[2]River Ecosystems Laboratory, Center for Alpine and Polar Environmental Research (ALPOLE), Ecole Polytechnique Fédérale de Lausanne (EPFL), Lausanne, Switzerland
[3]Biological and Environmental Sciences and Engineering Division (BESE), King Abdullah University of Science and Technology (KAUST), Thuwal, Saudi Arabia
[4]Department of Life Sciences and Medicine, Faculty of Science, Technology and Medicine, University of Luxembourg, Esch-sur-Alzette, Luxembourg

Supplementary Methods

A) DNA extraction protocol from alpine stream biofilms (rDNA)

Remark: Every time you open the tubes make sure that there is no liquid on the lids by applying a short spin

1. In a 1.5-ml tube add 10-20% (~300 ul) 0.1 mm Zirconium beads (Cole-Parmer 36270-62) per volume and 750 ml of Lysis buffer mixed with 0.5ul of RNase (100 mg/ml, Qiagen 19101)
2. Add 0.05 to 0.1 g and bead-beat at 6000 r/min, 2x 15sec-break 15sec (Precellys 24 homogenizer)
3. Incubate at 37 °C for 1 h with gentle agitation

4. Spin samples, add 5 ul Proteinase K (20 mg/ml, Fisher Scientific Cat.No. 25530049) and mix a few times
5. Incubate statically at 70 °C for 10 min
6. Centrifuge at 12.000 x g for 1 min and transfer all supernatant to a new 1.5 ml microtube
7. Spin samples and add 1 vol of Phenol:CHCl3:IAA (Fisher Scientific, 15593049)
8. Mix thoroughly and centrifuge at 13.000 x g for 10 min
9. Transfer aqueous phase into a new 1.5-ml tube and add 1 vol ml Chloroform – isoamyl alcohol mixture (Sigma, 25666)
10. Mix thoroughly and centrifuge at 13.000 x g for 5 min
11. Transfer supernatant to a new 2ml tube and then add 1/10th volume of 3M sodium acetate (pH 5.2) (Sigma S7899)
12. Add 0.7 volumes of ice-cold Isopropanol (Sigma I9516) and mix thoroughly
13. Precipitate DNA at -20 °C overnight
14. Centrifuge at 12.000 x g at 4 °C for 15 min
15. Remove supernatant and discard without disturbing the pellet
16. Wash 2 times with 0.4 ml of 70% EtOH and centrifuge at 13.000 g at 4 °C for 10 min
17. Air-dry the pellet, and elute with 100 ul RNase-free, DNase-free water (Qiagen 129112)
18. Let DNA pellet to dissolve o/n at 4 °C
19. Use 2 ul sample to quantify DNA using Qubit HS dsDNA (Invitrogen Q32854)

B) NCBI Accessions for *Polaromonas* genomes

**Name**	**AccessionID**

OUT1	GCF_001955735.1_ASM195573v1
DB1	GCF_000013865.1_ASM1386v1
DB2	GCF_000015505.1_ASM1550v1
DB3	GCF_000282655.1_Polaromonas.strCF318_v1.0
DB4	GCF_000688115.1_ASM68811v1
DB5	GCF_000709345.1_Polaromonas_sp.
DB6	GCF_001598235.1_ASM159823v1
DB7	GCF_002001015.1_ASM200101v1
DB8	GCF_002002705.1_ASM200270v1
DB9	GCF_002379085.1_ASM237908v1
DB10	GCF_002379095.1_ASM237909v1
DB11	GCF_003711205.1_ASM371120v1
DB12	GCF_009664225.1_ASM966422v1
DB13	GCF_012584515.1_ASM1258451v1
DB14	GCF_014641715.1_ASM1464171v1
DB15	GCF_015751795.1_ASM1575179v1
DB16	GCF_015752205.1_ASM1575220v1
DB17	GCF_015752225.1_ASM1575222v1
DB18	GCF_900103405.1_IMG-taxon_2636416056_annotated_assembly
DB19	GCF_900112285.1_IMG-taxon_2609459740_annotated_assembly
DB20	GCF_900116715.1_IMG-taxon_2615840640_annotated_assembly

Supplementary Note

Sloan model summary: The dispersal rate coefficient (m) and the goodness of fit of the beta distribution model (R^2) based on the Sloan neutral model analyses are indicated for New Zealand and Caucasus with respect to the metabarcoding information per amplicon sequence variant (ASV).

	16S rRNA gene amplicons		18S rRNA gene amplicons	
	R^2	*m*	R^2	*m*
New Zealand	-0.726	3.7E-04±4E-04	-0.204	4.3E-04±3E-04
Caucasus	-1.11	6.4E-04±5E-04	-0.527	0.57±0.31

Supplementary Figure Legends

Supplementary Figure 1. Glacier-fed streams from where epilithic and epipsammic biofilms were sampled.
Regions indicating the collection sites for the epilithic and epipsammic biofilms from (a) Caucasus and (b) Southern Alps. Relative abundance of prokaryotes (c) and eukaryotes (d) at the phylum and subdomain levels based on the sequencing of the 16S and 18S rRNA genes, respectively.

Supplementary Figure 2. Epilithic biofilm metagenomic profiles.
(a) Relative abundance profiles across the three domains of life: archaea, bacteria and eukaryotes in the epilithic biofilms, obtained from the sample metagenomes. Samples from the Southern Alps are indicated in red, while those from Caucasus are shown in blue. (b) Virome profile indicating the top 50 viruses. Scaled abundance from low (-2) to high (2) is indicated in the heatmap.

Supplementary Figure 3. Cross-domain interactions and adaptations of epilithic biofilms.
(a) Corrplot based on Spearman's correlation between pro- and eukaryotic MAGs aggregated at the phylum level. (b) Co-occurrence network of all MAGs across the Southern Alps in New Zealand and Caucasus in Russia. Each node represents a MAG, while the size represents the

degree centrality. The edges represent the positive coefficient of co-occurrence along with the corresponding betweenness centrality between the MAGs. Unconnected nodes represent MAGs with lower betweenness (< 0.5) compared to other MAGs. The color of the nodes represents the individual taxa, while the lines represent the edges connecting the nodes. The thickness of the lines indicates those edges with a betweenness greater than 0.5. Co-occurrence network constructed from pro- and eukaryotic MAGs found in (c) the Southern Alps (New Zealand) and (d) the Caucasus. The largest connected component of the co-occurrence network from (e) the Southern Alps (New Zealand) and (f) Caucasus GFSs are depicted.

Supplementary Figure 4. Extracellular enzyme genes based on lifestyle.
The classification at phylum and genus levels of MAGs identified as (a) heterotrophs, (b) phototrophs, or (c) those with 'unknown' trophic metabolisms are depicted, showing the abundance of genes encoding for extracellular enzymes. NA: unclassified genus; AG: α-1,4-glucosidase; BG: β-1,4-glucosidase; LAP: leucine aminopeptidase; NAG: β-1,4-N-acetylglucosaminidase; AP: acid (alkaline) phosphatase. (d) (c) Spearman's correlation analyses of overall eukaryote relative abundances with the CAZyme abundances. CAZymes include AA: auxilliary activities, CBM: carbohydrate-binding module , CE: carbohydrate esterases , GH: glycoside hydrolases, GT: glycosyltransferases, PL: polysaccharide lyases. FDR-adjusted *p*-values were estimated using the 'cor.mtest' function from the *corrplot* R package and are indicated by *, *i.e.*, * < 0.05, ** < 0.01, *** < 0.001.

Supplementary Figure 5. Comparison to public metagenomes reveals differential gene abundances.
Volcano plot indicating the total number of KOs (n = 9,335; total = 17,406) enriched in epilithic biofilms compared to 105 publicly available metagenomes. KO enrichment was assessed using DEseq2, where the adjusted *p*-value < 0.05 was considered to be significant.

Supplementary Data Legends

Supplementary Data 1. CAZyme abundances
Normalised abundances of the carbohydrate-active enzymes (CAZymes) across all samples. AA: auxilliary activities, CBM: non-catalytic carbohydrate-binding modules, CE:

carbohydrate esterases, GH: glycoside hydrolases, GT: glycosyltransferases, PL: polysaccharide lyases, and SLH: S-layer homology domain enzymes.

Supplementary Data 2. Public metagenomes
Metadata including ecosystems and location of the publicly-available metagenomes used for comparing Kyoto Encyclopedia of Genes and Genomes (KEGG) orthologs.

Supplementary Data 3. Enriched KEGG orthologs in epilithic biofilms.
KEGG orthology (KO) genes enriched in epilithic biofilms compared to other metagenomic datasets. Gene enrichment was assessed using DEseq2, where the adjusted p-value < 0.05 was considered to be significant.

Supplementary Data 4. COG functions enriched in GFS *Polaromonas* spp..
Clustered-orthologous genes (COG20) functions enriched in *Polaromonas* spp. compared to genomes available via RefSeq. Gene enrichment was assessed using DEseq2, where the adjusted p-value < 0.05 was considered to be significant.

Supplementary Data 5. Sample metadata.
Sample metadata including physico-chemical parameters such as pH, turbidity, conductivity, dissolved organic carbon, temperature, and CO_2 saturation.

Supplementary Data 6. Accession information.
NCBI sequence read archive (SRA) accession IDs for all samples used in the study including hyperlinks for each sample.

Supplementary Data 7. Osmotic stress genes
Gene counts for osmotic stress found in respective Phyla.

Supplementary figure 1. Sediment and epilithic biofilm sites

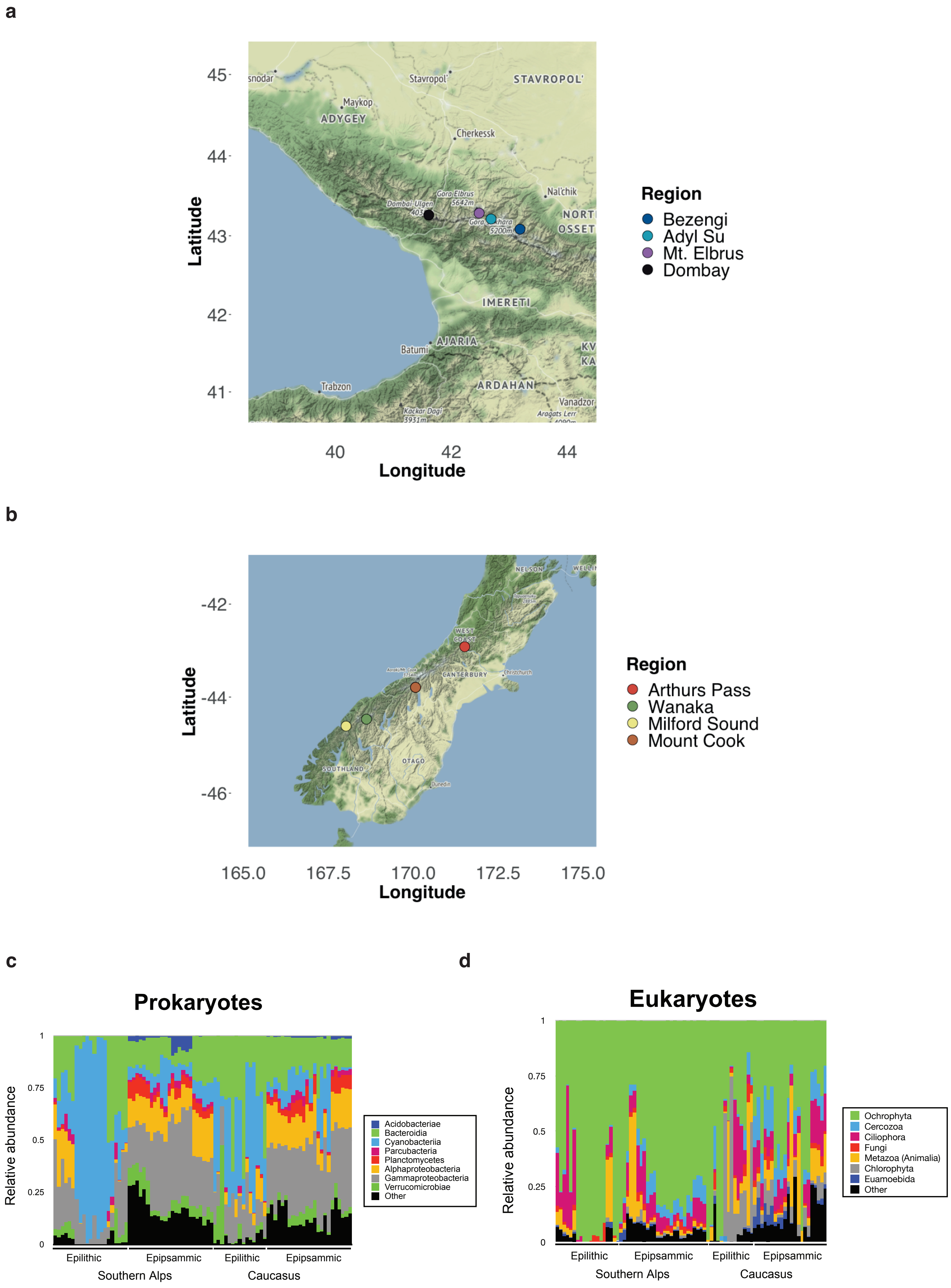

Supplementary figure 2. Epilithic biofilm metagenomic profiles

a

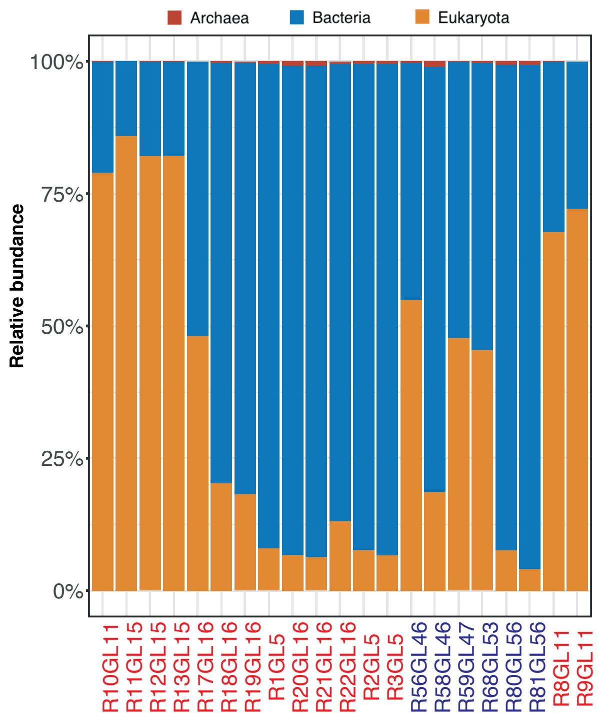

b

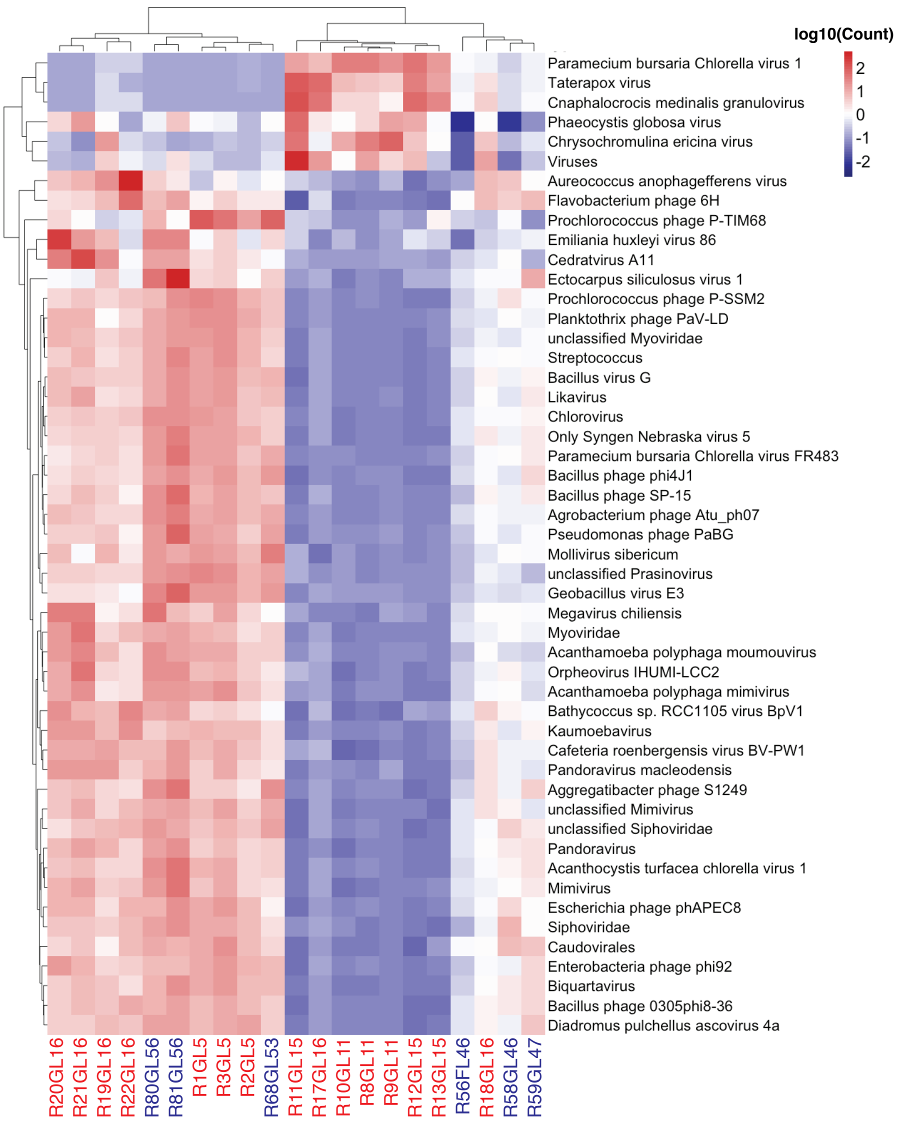

Supplementary figure 3. Cross-domain interactions and adaptations of epilithic biofilms

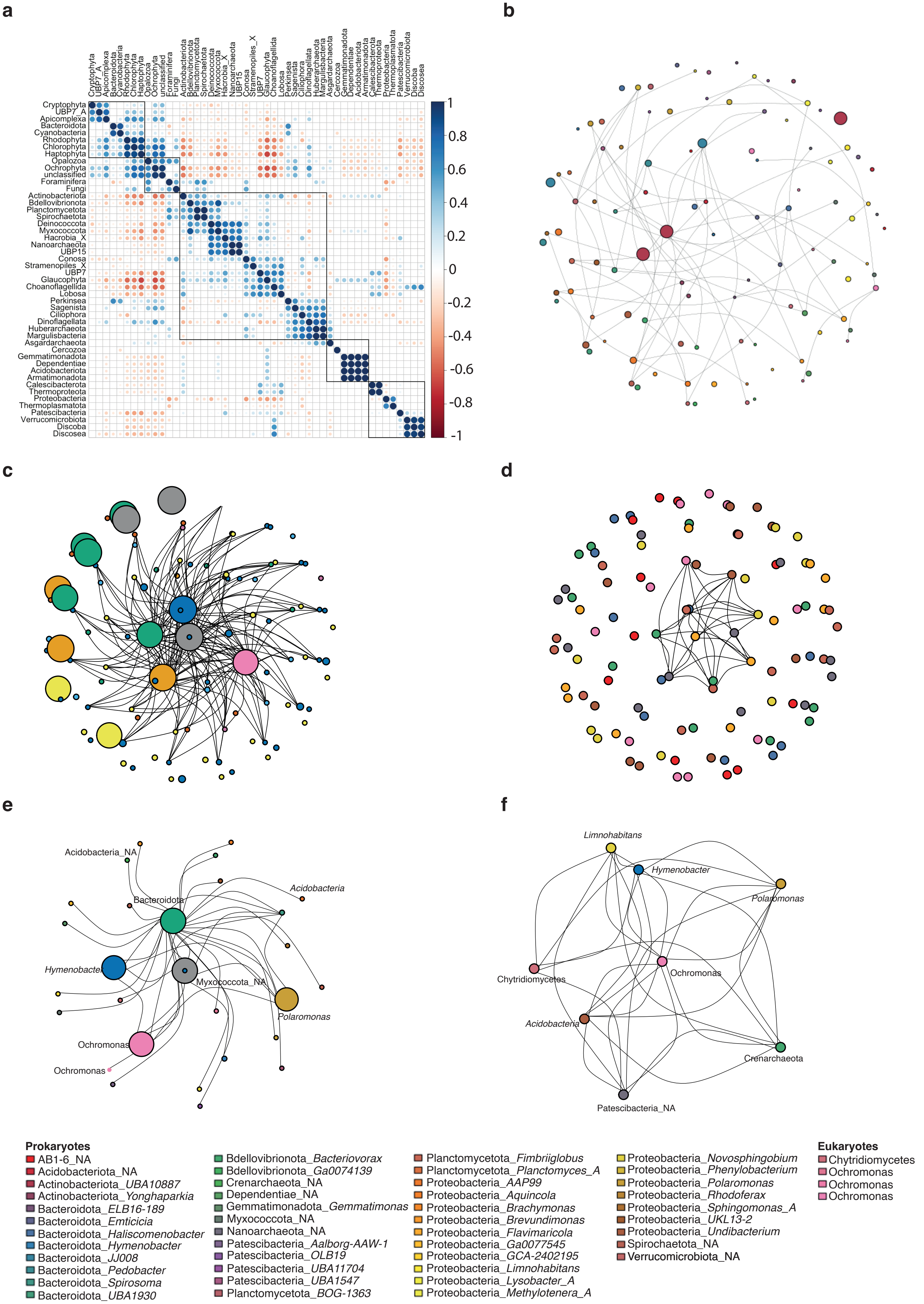

Supplementary figure 4. Extracellular enzyme genes based on lifestyle

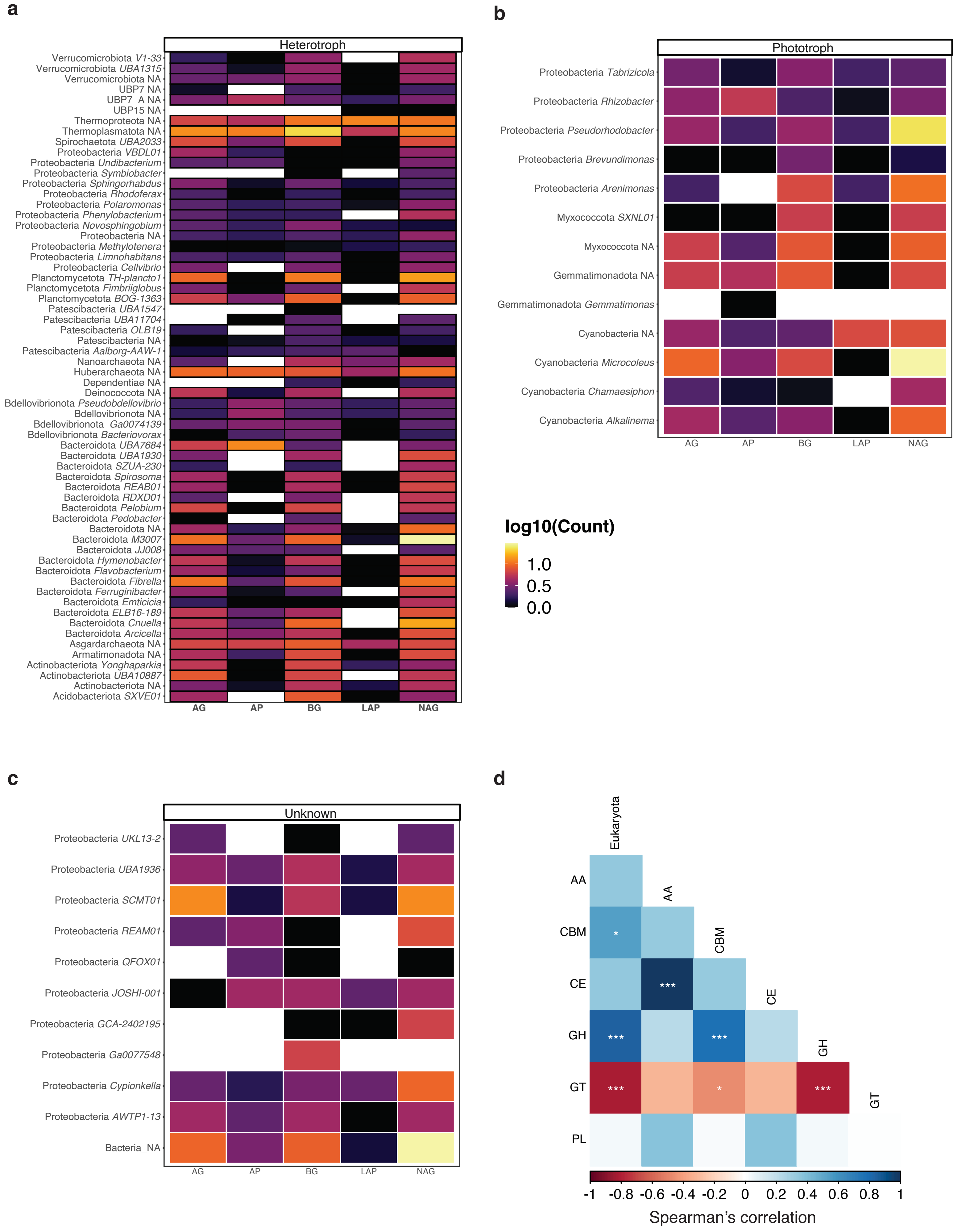

Supplementary figure 5. Comparison to public metagenomes reveals differential gene abundances

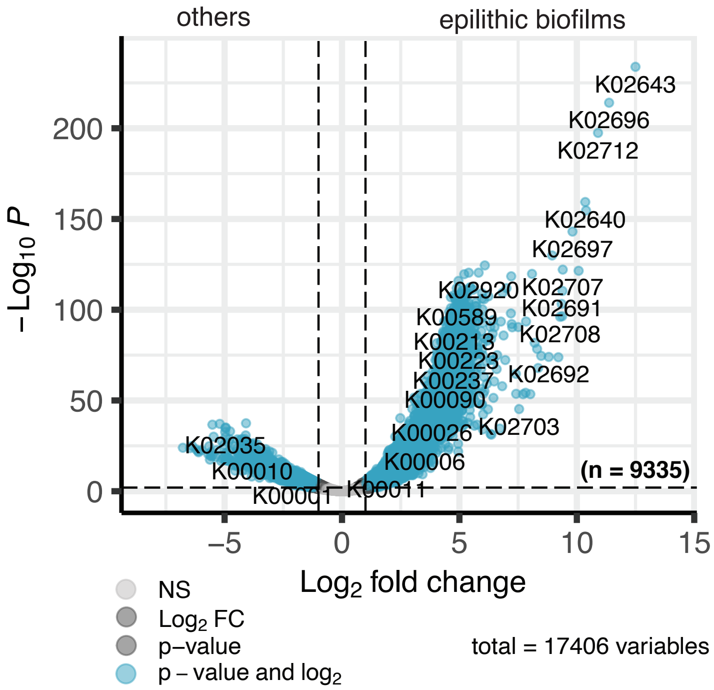

Supplement: Supplementary file 1 — Supplementary Information [file 41467_2022_29914_MOESM1_ESM.pdf]
