# Supplementary material for: Genomic and metabolic adaptations of biofilms to ecological windows of opportunity in glacier-fed streams
Source: Nat Commun. 2022 Apr 20;13:2168. doi: 10.1038/s41467-022-29914-0 (PMC9021309; doi:10.1038/s41467-022-29914-0)
Supplement: Supplementary file 3 — Description of Additional Supplementary Information [file 41467_2022_29914_MOESM3_ESM.pdf]

Description of Additional Supplementary Files

Title: Supplementary Data 1

Description: CAZyme abundances Normalised abundances of the carbohydrate-active enzymes (CAZymes) across all samples. AA: auxilliary activities, CBM: non-catalytic carbohydrate-binding modules, CE: carbohydrate esterases, GH: glycoside hydrolases, GT: glycosyltransferases, PL: polysaccharide lyases, and SLH: S-layer homology domain enzymes.

Title: Supplementary Data 2

Description: Public metagenomes Metadata including ecosystems and location of the publicly-available metagenomes used for comparing Kyoto Encyclopedia of Genes and Genomes (KEGG) orthologs.

Title: Supplementary Data 3

Description: Enriched KEGG orthologs in epilithic biofilms. KEGG orthology (KO) genes enriched in epilithic biofilms compared to other metagenomic datasets. Gene enrichment was assessed using DEseq2, where the adjusted p-value < 0.05 was considered to be significant.

Title: Supplementary Data 4

Description: COG functions enriched in GFS Polaromonas spp.. Clustered-orthologous genes (COG20) functions enriched in Polaromonas spp. compared to genomes available via RefSeq. Gene enrichment was assessed using DEseq2, where the adjusted p-value < 0.05 was considered to be significant.

Title: Supplementary Data 5

Description: Sample metadata. Sample metadata including physico-chemical parameters such as pH, turbidity, conductivity, dissolved organic carbon, temperature, and CO_2 saturation.

Title: Supplementary Data 6

Description: Accession information. NCBI sequence read archive (SRA) accession IDs for all samples used in the study including hyperlinks for each sample.

Title: Supplementary Data 7

Description: Osmotic stress genes Gene counts for osmotic stress found in respective Phyla.
