# Supplementary material for: Genomic and metabolic adaptations of biofilms to ecological windows of opportunity in glacier-fed streams
Source: Nat Commun. 2022 Apr 20;13:2168. doi: 10.1038/s41467-022-29914-0 (PMC9021309; doi:10.1038/s41467-022-29914-0)
Supplement: Supplementary file 11 — Reporting Summary [file 41467_2022_29914_MOESM11_ESM.pdf]

nature portfolio

Corresponding author(s): Tom Battin and Paul Wilmes

Last updated by author(s): 03/21/2022

Reporting Summary

Nature Portfolio wishes to improve the reproducibility of the work that we publish. This form provides structure for consistency and transparency in reporting. For further information on Nature Portfolio policies, see our Editorial Policies and the Editorial Policy Checklist.

Statistics

For all statistical analyses, confirm that the following items are present in the figure legend, table legend, main text, or Methods section.

n/a	Confirmed	
☐	☒	The exact sample size (*n*) for each experimental group/condition, given as a discrete number and unit of measurement
☐	☒	A statement on whether measurements were taken from distinct samples or whether the same sample was measured repeatedly
☐	☒	The statistical test(s) used AND whether they are one- or two-sided<br>*Only common tests should be described solely by name; describe more complex techniques in the Methods section.*
☐	☒	A description of all covariates tested
☐	☒	A description of any assumptions or corrections, such as tests of normality and adjustment for multiple comparisons
☐	☒	A full description of the statistical parameters including central tendency (e.g. means) or other basic estimates (e.g. regression coefficient) AND variation (e.g. standard deviation) or associated estimates of uncertainty (e.g. confidence intervals)
☐	☒	For null hypothesis testing, the test statistic (e.g. *F*, *t*, *r*) with confidence intervals, effect sizes, degrees of freedom and *P* value noted<br>*Give P values as exact values whenever suitable.*
☒	☐	For Bayesian analysis, information on the choice of priors and Markov chain Monte Carlo settings
☐	☒	For hierarchical and complex designs, identification of the appropriate level for tests and full reporting of outcomes
☐	☒	Estimates of effect sizes (e.g. Cohen's *d*, Pearson's *r*), indicating how they were calculated

Our web collection on statistics for biologists contains articles on many of the points above.

Software and code

Policy information about availability of computer code

Data collection

The data was collected using the below listed methods.

Sample collection
We sampled a total of eight GFSs from the New Zealand Southern Alps and the Russian Caucasus in early- and mid-2019, respectively, for a total of 27 epipsammic samples taken from sandy sediments and 21 epilithic biofilm samples from boulders adjacent to the epipsammic samples (Supp. Table 5). Epipsammic samples were collected from each GFS by first identifying three patches within a reach of ~5-10 m. From each patch, sandy sediments were taken from the <5 cm surface of the streambed with a flame-sterilized metal scoop and sieved to retain the 250 µm to 3.15 mm size fraction. While three epipsammic samples were taken from each stream, epilithic samples were taken opportunistically from up to three boulders per reach (Supp. Table 5). Epilithic biofilms were sampled using a sterilized metal spatula. All samples were immediately flash-frozen in liquid nitrogen in the field and transported and stored frozen pending DNA extraction. Streamwater turbidity, conductivity, temperature, and pH were measured in situ during the sampling (Supplementary Data 2).

DNA extraction and purification
A previously established protocol69 was used to extract DNA from all samples. Briefly, 5 g of epipsammic and 0.05-0.1 g of epilithic biofilm were subjected to a phenol:chloroform-based extraction and purification method. The differential input volume for the DNA extractions were established to account for the differences in biomass between the epipsammic and epilithic biofilms. The samples were treated with a lysis buffer containing SDS along with 0.1 M Tris-HCl pH 7.5, 0.05 M EDTA pH 8, 1.25% SDS and RNase A (10 µl: 100 mg/ml). The samples were vortexed and incubated at 37 °C for 1 h. Proteinase K (100 µl; 20 mg/ml) was subsequently added and further incubated at 70 °C for 10 min. Samples were purified once with phenol/chloroform/isoamyl alcohol (ratio 25:24:1, pH 8) and the supernatant was subsequently extracted with a 24:1 ratio chloroform/isoamyl alcohol. Linear polyacrylamide (LPA) was used along with sodium acetate and ice-cold isopropanol for precipitating that DNA overnight at -20 °C. For epilithic biofilms, the entire protocol was adapted to a smaller scale due to the availability of higher DNA concentrations compared to sediment. The former was treated with 0.75 ml of lysis buffer (instead of 5 ml for sediment) and all subsequent volumes of reagents were adapted accordingly (see supplementary material). Furthermore, a mechanical lysis step of bead-beating was necessary along with a lysis buffer to facilitate DNA release from the more developed epilithic biofilms. Due to the higher DNA

yields, the addition of LPA was omitted from the DNA precipitation step. DNA quantification was performed for all samples with the Qubit dsDNA HS kit (Invitrogen).

Metabarcoding library preparation and sequencing
The prokaryotic 16S rRNA gene metabarcoding library preparation was performed as described in Fodelianakis et al.70, targeting the V3-V4 hypervariable region of the 16S rRNA gene with the 341F/785R primers and following Illumina guidelines for 16S metagenomic library preparation for the MiSeq system. The eukaryotic 18S rRNA gene metabarcoding library preparation was performed likewise but using the TAReuk454F-TAReukREV3 primers to target the 18S rRNA gene V4 loop71. Samples were sequenced using a 300-bp paired-end protocol partly in the Genomic Technologies Facility of the University of Lausanne (27 epipsammic samples) and partly at the Biological Core Lab of the King Abdullah University of Science and Technology (21 epilithic samples).

Whole-genome shotgun libraries and sequencing
All epilithic biofilm DNA samples underwent random shotgun sequencing following library preparation using the NEBNext Ultra II FS library kit. Briefly, 50 ng of DNA was used for constructing metagenomic libraries under 6 PCR amplification cycles, following enzymatic fragmentation of the input DNA for 12.5 mins. The average insert size of the libraries was 450 bp. Qubit (Invitrogen) was used to quantify the libraries followed by quality assessment using the Bioanalyzer from Agilent. Sequencing was performed at the Functional Genomics Centre Zurich on a NovaSeq (Illumina) using a S4 flowcell.

Data analysis

Metabarcoding analyses.
The 16S rRNA gene metabarcoding data were analysed using a combination of Trimmomatic v0.32 and QIIME2 v2021.4 as described in Fodelianakis et al.86, with the exception that here the latest SILVA database90 v138.1 was used for taxonomic classification of 16S rRNA and 18S rRNA gene amplicons. Non-bacterial ASVs including those affiliated to archaea, chloroplasts and mitochondria were discarded from the 16S rRNA amplicon dataset in all downstream analyses. ASVs observed only once were removed from both 16S rRNA and 18S rRNA amplicon datasets. Diversity analyses were performed in R using the vegan v2.5.7 and metacoder92 v0.3.5 packages. For non-metric multidimensional scaling (nMDS) and distance-based redundancy (db-RDA) analyses data were log(x+1) transformed and the capscale and ordiR2step (backwards direction, 200 permutations) functions from vegan were used. To test for a source-sink hypothesis from epipsammic to epilithic, the Sloan's Neutral Community Model27 was used based on the R implementation developed by Burns et al.93.

Metagenomic preprocessing, assembly, binning, and analyses
For processing metagenomic sequence data, we used the Integrated Meta-omic Pipeline (IMP)78 workflow to process paired forward and reverse reads using version 3.0 (commit# 9672c874; available at https://git-r3lab.uni.lu/IMP/imp3), as previously described79. IMP's workflow includes pre-processing, assembly, genome reconstructions and additional functional analysis of genes based on custom databases in a reproducible manner. Briefly, adapter trimming is followed by an iterative assembly using MEGAHIT v1.2.980. Concurrently, MetaBAT2 v2.12.181 and MaxBin2 v2.2.782 are used for binning in addition to an in-house method established previously79 for reconstructing metagenome-assembled genomes (MAGs). Binning was completed by selecting a non-redundant set of MAGs using DASTool83 based on a score threshold of 0.7. The quality of the MAGs was assessed using CheckM v1.1.384, while taxonomy was assigned using the GTDB-toolkit v1.4.185.
For the downstream analyses including identification of viruses, VIBRANT v1.2.186 was used on the metagenomic assemblies. The output from this was used to identify the viral taxa using vConTACT2 v0.9.2287. Independently, the viral contigs were also validated using CheckV v0.7.088. To estimate the overall abundances of eukaryotes along with prokaryotes including archaea, we used EUKulele v1.0.589 with both the MMETSP and the PhyloDB databases, run separately, to confirm the detected eukaryotic profiles. To understand the overall metabolic and functional potential of the metagenome and reconstructed MAGs we used MANTIS90. Additionally, we used METABOLIC v4.091, metabolisHMM v2.2192, and Lithogenie from MagicLamp v1.0 (https://github.com/Arkadiy-Garber/MagicLamp) to identify metabolic and biogeochemical pathways relevant for determining nutritional phenotypes of all MAGs along with the 'anvi-estimate-metabolism' function from anvi'o93. This information was manually validated based on the different tools to identify which MAGs encode for the respective pathways. Subsequently, to determine the growth rates of prokaryotes, we used codon usage statistics for detecting optimization of genes that are highly expressed, as an indicator of maximal growth rates with gRodon v1.094. All the parameters, databases, and relevant code for the analyses described above are openly available at https://git-r3lab.uni.lu/susheel.busi/nomis_pipeline and included in the Code availability section.

Eukaryote assembly and binning
To obtain eukaryotic MAGs, an alternate, custom pipeline (https://github.com/Mass23/NOMIS_ENSEMBLE/tree/coassembly) was established for coassembling the twenty-one epilithic biofilm sequence data with subsequent binning. Individual samples were first preprocessed similar to the workflow used in IMP, i.e., using FastP v0.20.095. Subsequently, the reads were deduplicated to avoid overlap and enhance computation efficiency using clumpify.sh from the BBmap suite v38.7996. Thereafter, any reads mapping to bacteria or viruses were removed by filtering the reads against a Kraken2 v2.0.9beta97 maxikraken database available at https://lomanlab.github.io/mockcommunity/mc_databases.html. Only reads that were unknown or mapping to eukaryotes were retained and concatenated. This was followed by another round of deduplication using clumpify.sh. The concatenated reads were assembled using MEGAHIT v1.2.7 with the following options: --kmin-1pass -m 0.9 --k-list 27,37,47,57,67,77,87 --min-contig-len 1000. Following assembly, EukRep v0.6.798 was used for retrieving eukaryotic contigs with a minimum length of 2000 bp and the '-m strict' flag. These contigs were used for binning into MAGs as described herein.
Eukaryotic MAGs were binned using CONCOCT v1.1.099. To do this, coverages were estimated for the contigs by mapping the reads of all samples against the contigs using the coverm v0.6.1 (https://github.com/wwood/CoverM) to generate bam files. These files were then used to generate a table with coverage depth information per sample. The protein coding genes of the MAGs was predicted with MetaEuk v4.a0f584d100 with their in-house database made with MERC, MMETSP and Uniclust50 (http://wwwuser.gwdg.de/~compbiol/metaeuk/). The annotation was then subsequently done with eggNOG-mapper v2.1.0101. The completeness and contamination of the MAGs were assessed with Busco v5.0.0102 and the eukaryotic lineage (255 genes). We determined their taxonomy by comparing the results of the EUKulele v1.0.389 and EukCC v0.3103 along with homology comparisons with publicly available genomes not included in the previous tools by protein BLAST v2.10.0104.

Co-occurrence interaction networks
Co-occurrence networks between the pro- and eukaryotic MAGs were constructed using an average of the distance matrices created from SparCC, Spearman's correlation and SpiecEasi where the networks were constructed using the 'Meinshausen and Bühlmann (mb)' method. Nodes with fewer than two degrees were discarded to identify cliques with three or more interactions, while negative edges were removed to visualize only mutualistic relationships. The matrix was visualised using the igraph v1.1.2 R package. The largest component from the overall co-occurrence network was determined using the components module of the igraph package. Null model hypothesis was tested by assessing the distribution of the node degree and the respective probabilities of the occurrence network against those simulating the Erdos-Renyi,

Barabasi-Albert, Stochastic-block null models108.

Phylogenomics and pangenomes
For the pangenome analyses, we collected all the bins taxonomically identified as Polaromonas spp. and used the pangenome workflow described by Meren et al. (http://merenlab.org/2016/11/08/pangenomics-v2/) using anvi'o, along with NCBI refseq genomes for comparison and an outgroup from the closely related Rhodoferax genus. The choice of Polaromonas spp. was based on its high abundance and prevalence within the epilithic biofilms. The accession IDs from the reference genomes obtained from NCBI are provided in the supplementary material. The pangenome was run using the --min-bit 0.5, --mcl-inflation 10 and --min-occurence 2 parameters, excluding the partial gene calls. A phylogenomic tree was built using MUSCLE v3.8.1551110 and FastTree2 v2.1.10111 on all single-copy gene clusters in the pangenome that were present in at least 30 genomes and had a functional homogeneity index below 0.9, and geometric homogeneity index above 0.9. The phylogenomic tree was used to order the genomes, the frequency of gene clusters (GC) to order the GC dendrogram. A phylogenomic bacterial tree of life containing the 47 high-quality MAGs along with 264 NCBI bacterial genomes was built based on a set of 74 single-copy genes using the GToTree v1.5.51112 pipeline with the -D parameter, allowing to retrieve taxonomic information for the NCBI accessions. Briefly, HMMER3 v3.3.2113 was used to retrieve the single-copy genes after gene-calling with Prodigal v2.6.3114 and aligned using TrimAl v1.4.rev15115. The entire workflow is based on GNU Parallel v20210222116.

Data analyses and figures
Figures for the study including visualizations derived from the taxonomic and functional components, were created using version 3.6 of the R statistical software package117. The maps indicating the collection sites were generated using the ggmap v3.0.0 package in R. KEGGDecoder119 was used to assess enriched KEGG orthology (KO) IDs in comparison to 105 publicly available metagenome sampled in various ecosystems at a global scale (Supp. Tables 3 and 6), which were processed using the IMP workflow. DESeq2120 with FDR-adjustments for multiple testing were used to assess KOs significantly enriched in the GFS metagenomes compared to this comparison dataset. The volcano plot highlighting the significant KOs was generated using the EnhancedVolcano121 R package. Figures from metabarcoding data were also generated in Rv3.6 using the ggplot2122 package and were further annotated graphically using Inkscape.

Code availability
The detailed code used for the downstream functional and growth analyses is available at https://git-r3lab.uni.lu/susheel.busi/nomis_pipeline and https://doi.org/10.5281/zenodo.6372573. The custom pipeline for eukaryote analyses can be found here: https://github.com/Mass23/NOMIS_ENSEMBLE/tree/coassembly. Subsequent binning and manual refinement of eukaryotic MAGs was done as described here: https://git-r3lab.uni.lu/susheel.busi/nomis_pipeline/-/blob/master/workflow/notes/MiscEUKMAGs.md.

For manuscripts utilizing custom algorithms or software that are central to the research but not yet described in published literature, software must be made available to editors and reviewers. We strongly encourage code deposition in a community repository (e.g. GitHub). See the Nature Portfolio guidelines for submitting code & software for further information.

Data

Policy information about availability of data

All manuscripts must include a data availability statement. This statement should provide the following information, where applicable:

- Accession codes, unique identifiers, or web links for publicly available datasets
- A description of any restrictions on data availability
- For clinical datasets or third party data, please ensure that the statement adheres to our policy

Data availability
Raw sequencing data samples and the MAGs are available at NCBI's sequence read archive under BioProject accession PRJNA733707. The Biosample accession IDs and the metadata associated with each sample are listed under Supp. Data 6. A snippet of the results and source data generated and used in this study have been deposited in Zenodo at https://doi.org/10.5281/zenodo.5545722.

Databases used in the current study include the following:
1. maxikraken database: https://lomanlab.github.io/mockcommunity/mc_databases.html
2. MetaEuk databases: http://wwwuser.gwdg.de/~compbiol/metaeuk
3. SILVA database: https://www.arb-silva.de/documentation/release-1381/
4. MMETPS and PhyloDB databases: https://eukulele.readthedocs.io/en/latest/databaseandconfig.html

Field-specific reporting

Please select the one below that is the best fit for your research. If you are not sure, read the appropriate sections before making your selection.

☐ Life sciences ☐ Behavioural & social sciences ☒ Ecological, evolutionary & environmental sciences

For a reference copy of the document with all sections, see nature.com/documents/nr-reporting-summary-flat.pdf

Ecological, evolutionary & environmental sciences study design

All studies must disclose on these points even when the disclosure is negative.

Study description: The study targets the characterisation of epilithic biofilms found glacier-fed streams (GFSs) using both amplicon and whole genome shotgun sequencing methodologies. In GFSs, ecological windows of opportunities allow complex microbial biofilms to develop and transiently form the basis of the food web, thereby controlling key ecosystem processes. Here, using high-resolution metagenomics,

March 2021

	we unravel strategies that allow epilithic biofilms to seize this opportunity in an ecosystem otherwise characterized by harsh environmental conditions.
Research sample	27 epipsammic samples taken from sandy sediments and 21 epilithic biofilm samples from boulders adjacent to the epipsammic samples collected from GFSs as described above are meant to serve as a representation of the microbial community within GFSs. Metagenomic sequencing was subsequently used to deeply characterise the eplithic biofilms identifying gene- and metabolic-centric adaptations to the nutrient-limited GFSs.
Sampling strategy	Based on expeditions in the summer, when glaciers are typically melting and GFSs are free-flowing, we sampled a total of eight GFSs from the New Zealand Southern Alps and the Russian Caucasus in early- and mid-2019, respectively. Epipsammic samples were collected from each GFS by first identifying three patches within a reach of ~5-10 m. From each patch, sandy sediments were taken from the <5 cm surface of the streambed with a flame-sterilized metal scoop and sieved to retain the 250 µm to 3.15 mm size fraction. While three epipsammic samples were taken from each stream, epilithic samples were taken opportunistically from up to three boulders per reach (Supp. Table 5). Epilithic biofilms were sampled using a sterilized metal spatula. All samples were immediately flash-frozen in liquid nitrogen in the field and transported and stored frozen pending DNA extraction.<br><br>Number of samples were selected based on the availability of epilithic biofilms, per reach, per stream. We collected true biological replicates within each stream, from independent patches in each stream. Given the broad range of glaciers, including geographical, and spatial distributions, we believe the number of samples is representative of epilithic biofilms in GFSs. Additionally, our sampling efforts took into account individual metrics such as latitude, longitude, elevation and other physico-chemical data which are available in Supplementary Data 5.
Data collection	Streamwater turbidity, conductivity, temperature, and pH were measured in situ during the sampling (Supplementary Data 2), by the expedition team members: Michail Styllas, Matteo Tolosano, Vincent de Staercke and Martina Schon.<br><br>We measured stream temperature, pH, and dissolved oxygen in situ using a WTW Multiparameter portable meter (MultiLine® Multi 3630 IDS), conductivity using a WTW - IDS probe (TetraCon® 925). Stream water turbidity was measured using a PME Cyclops-7 Logger with a logging rate of 1 min, and values were averaged over the duration of the sampling. Samples for dissolved organic carbon (DOC) were collected into acid-washed precombusted glass vials, stored in the dark at 4 °C, and analyzed using a Sievers M5310c TOC Analyzer (GE Analytical Instruments) (accuracy: ±2%, precision: <1%, detection limit: 22 µg C/L).
Timing and spatial scale	As described above, epipsammic samples were collected from each GFS by first identifying three patches within a reach of ~5-10 m. While three epipsammic samples were taken from each stream, epilithic samples were taken opportunistically from up to three boulders per reach (Supp. Table 5). The samples were collected from the Southern Alps in New Zealand between January-February 2019, and from the Caucasus in Russia in September-October, 2019. Individual sample metrics such as latitude, longitude, elevation and other physico-chemical data are available in Supplementary Data 5.
Data exclusions	No data were excluded from the analyses. However, due to very poor DNA concentrations from the Epipsammic biofilm samples, whole genome shotgun libraries were unsuccessful and therefore their respective metagenomic sequence data was not part of the study.
Reproducibility	To ensure maximum reproducibility, samples were collected in triplicates and extracted simultaneously. All necessary protocols, including the code for the extensive data analyses and figure generation are provided. Appropriate blanks/controls were established during the DNA extraction, library preparation and sequencing phases.
Randomization	During the sampling process, epipsammic samples were collected from three random patches, within the limits of safety and feasibility. During the extraction processes, random sets of both epipsammic and epilithic samples were extracted to reduce any potential batch effects. Sample collected from the benthic sediment were allocated into the "epipsammic" group, whereas those biofilms found on rocks embedded immediately adjacent within the GFSs were collected to be part of the "epilithic" group. Covariate data for each of the samples is controlled for, by collecting adjacent epipsammic and epilithic biofilms ensuring similar physico-chemical parameters.
Blinding	Given the descriptive nature of our work, blinding was not necessary. Having said that, the DNA extractions were performed based on a sample number scheme, thus blinding the technicians to the exact origins of the samples. This scheme was maintained during the sequence analyses and post-processing steps.

Did the study involve field work? ☒ Yes ☐ No

Field work, collection and transport

Field conditions	All the relevant parameters such as temperature, turbidity, glacier area, etc. are detailed in Supplementary Data 5
Location	The Southern Alps in New Zealand and the Caucasus in Russia. The elevation, latitude and longitude for each GFS are listed in Supplementary Data 5
Access & import/export	We have worked with both the local and national authorities to assure that we had all required sampling and export permission. In New Zealand, we had the permissions from the various Maori tribes, while in Russia, we worked with the Russian Academy of Sciences to get clearance from the respective ministries.<br><br>In New Zealand, we obtained the necessary authorization to undertake research and collect and export our samples form the Department of Conservation (Te Papa Atawhai), under Research and samples collection authorization 72437-Res, issued on 11/01/2019.<br><br>In Russia, research and sampling authorization was provided through our official collaboration with the Institute of Geography of the

	Russian Academy of Sciences (IGRAS, Dr Olga Solomina), while the exportation permit for the samples was provided by the Federal Service for Supervision of Natural Resources (21/10/2019), upon specific demand from IGRAS.
Disturbance	We did not disturb the environment by sampling sediments and water.

Reporting for specific materials, systems and methods

We require information from authors about some types of materials, experimental systems and methods used in many studies. Here, indicate whether each material, system or method listed is relevant to your study. If you are not sure if a list item applies to your research, read the appropriate section before selecting a response.

Materials & experimental systems

n/a	Involved in the study
☒	☐ Antibodies
☒	☐ Eukaryotic cell lines
☒	☐ Palaeontology and archaeology
☒	☐ Animals and other organisms
☒	☐ Human research participants
☒	☐ Clinical data
☒	☐ Dual use research of concern

Methods

n/a	Involved in the study
☒	☐ ChIP-seq
☒	☐ Flow cytometry
☒	☐ MRI-based neuroimaging
